# Diabetic Ketoacidosis: Considerations and Residual Controversies in Management After the 2024 ADA, EASD, JBDS, AACE, and DST Joint Consensus

**DOI:** 10.2174/0118715303374972250407065308

**Published:** 2026-04-18

**Authors:** A. Ciafardini, W. Vena, N. Betella, S. Pigni, M. Mirani, V.M. Altieri, G. Mazziotti, A.G. Lania, A.C. Bossi

**Affiliations:** 1 Department of Biomedical Sciences, Humanitas University, Via Rita Levi Montalcini 4, 20090, Milan, Pieve Emanuele, Italy;; 2 Diabetes Center, Humanitas Gavazzeni Institute, Via M. Gavazzeni 21, 24100, Bergamo, Italy;; 3 Endocrinology, Diabetology and Medical Andrology Unit, IRCCS Humanitas Research Hospital, Via Manzoni 56, 20089, Milan, Rozzano, Italy;; 4 Department of Medicine and Health Sciences “V.Tiberio”, University of Molise, 88100 Campobasso, Italy;; 5 Department of Urology, Humanitas Gavazzeni, 24125, Bergamo, Italy

**Keywords:** Diabetic ketoacidosis, euglycemic diabetic ketoacidosis, diabetes mellitus, ketoacidosis treatment, pathogenesis, clinical onset

## Abstract

Diabetic ketoacidosis (DKA) is the most serious and life-threatening complication of Diabetes Mellitus (DM), characterized by the triad of hyperglycemia, ketonemia, and anion gap metabolic acidosis. DKA is more common in young people with type 1 diabetes (T1D) but can also occur in patients with type 2 diabetes (T2D) and in pregnant women with pregestational T1D or T2D or gestational DM. Moreover, DKA may be a rare complication of immune check-point inhibitor therapy. Euglycemic DKA (eDKA) is a variant of DKA with normal or minimally elevated serum glucose associated with using sodium-glucose cotransporter-2 (SGLT2) inhibitors, a class of anti-hyperglycemic medications. Prompt identification of DKA in the emergency setting is mandatory, and the management of its critical aspects and its possible underlying precipitating factors are often life-changing choices for patients. Despite diagnostic and therapeutic improvements, DKA still stands as one of the main causes of morbidity and mortality in DM individuals. Recently, an inter-society consensus report has been published to provide up-to-date knowledge on DKA. Nevertheless, controversies concerning the clinical management of this acute complication of DM remain to be unfolded and high-quality evidence is lacking in concern to solve such critical aspects. This narrative review aims to explore and discuss DKA, its epidemiology, pathogenesis, diagnosis, clinical onset, and treatment, highlighting some of the main remaining open controversies.

## 1 INTRODUCTION

Diabetic ketoacidosis (DKA) is the most serious and life-threatening complication of diabetes mellitus (DM), often requiring critical care admission. DKA is characterized by the triad of 1) hyperglycemia, 2) ketonemia, and 3) anion gap metabolic acidosis [1, 2]. Euglycemic DKA (eDKA) is a variant of DKA characterized by minimally elevated serum glucose associated with conditions such as pregnancy, decreased caloric intake, and sodium-glucose cotransporter-2 (SGLT2) inhibitors. While more common in young-individuals with type 1 diabetes (T1D), DKA may also occur in those with type 2 diabetes (T2D) [3]. Despite advances in diagnostic techniques, medical treatments, and technological resources, DKA continues to be a significant cause of morbidity and death among patients with DM. Recently, a consensus report from the American Diabetes Association (ADA), the European Association for the Study of Diabetes (EASD), the Joint British Diabetes Societies for Inpatient Care (JBDS), the American Association of Clinical Endocrinology (AACE), and the Diabetes Technology Society (DTS) has been published to provide current information on the epidemiology, pathophysiology, clinical manifestations, and guidelines for the diagnosis, treatment, and prevention of DKA [4]. Herein, we review the epidemiology, pathogenesis, diagnosis, and treatment of DKA, highlighting unresolved controversies.

## 2 HISTORY & VARIANTS

In 1828, August W. von Stosch reported the first clinical description of diabetic coma in an adult patient with severe polydipsia, polyuria, and significant glucosuria, followed by progressive mental decline and death [5]. Subsequently, numerous case reports have described patients with newly diagnosed or established DM presenting similar symptoms [6-8]. In 1874, Adolf Kussmaul reported several episodes of diabetic coma characterized by deep, rapid, and labored breathing [9, 10]. Shortly thereafter, it was discovered that the urine of most patients with diabetic coma contained acetoacetic acid and β-hydroxybutyric acid [11]. However, the first comprehensive description of diabetic ketoacidosis (DKA) is attributed to Dr. J. Dreschfeld, who presented it in 1886 during a lecture at the Royal College of Physicians during a lecture on diabetic coma [12]. Before the discovery of insulin in 1922 by Dr. Banting and Best, diabetic coma was universally fatal [13]. Just one year later, after insulin’s discovery, the same authors described the case of a 14-year-old boy with DKA admitted to the Toronto General Hospital and treated with insulin with prompt improvement and recovery [13]. In 1945, H. Root in Boston described that the mortality rate associated with DKA fell dramatically to 12% by 1940 and to 1.6% by 1945 using high doses of insulin [13]. It was not until 1971, when Roger Unger described DKA as a disorder involving insulin and glucagon ratio, that its pathophysiology became better understood [14]. In 1973 Munro *et al*. published the first case series of patients with DKA despite normal or minimally elevated serum glucose levels termed eDKA [14]. This condition gained attention in 2015 when the US Food and Drug Administration (FDA) and the European Medicines Agency (EMA) issued warnings linking SGLT-2 inhibitors use to eDKA predisposition [14].

## 3 EPIDEMIOLOGY

Over the last few decades, the incidence of diagnosed DM has been up to 3-fold higher (10.1% per 1000) among adults aged 45 and older compared to the younger population. However, during the period 2002-2018, the overall incidence of DKA in both T1D and T2D increased significantly among US children and adolescents [15]. DKA occurs more commonly in patients with T1D and may be the initial presentation of T1D in approximately 25-40% of all cases [16]. Among children and adolescents with T1D, the ADA estimates the risk of DKA to be around 1-10 per 100 person-years [17]. During the early years of the 21^st^ century, the reported incidence of DKA among adults with T1D in regions such as Europe, the USA, and Israel ranged from 0 to 56 cases per 1000 person-years. Conversely, a study carried out in China between 2010 and 2012 revealed an unusually high rate of 263 cases per 1000 person-years [18]. Interestingly, up to one-third of cases of DKA observed in clinical practice occur in subjects with T2D [19, 20]. Notably, up to 50% of all Black/African American diabetics presenting with DKA are diagnosed with the so-called “ketosis-prone diabetes” (KPD), which is a variant of DM exhibiting characteristics of both T1D and T2D, typically presenting with DKA or unprovoked ketosis. KPD is estimated to affect nearly 15% of all hospitalized diabetics in Africa [20].

DKA is responsible for up to 1% of emergency department visits among all adult DM patients and up to 1 in 4 hospitalizations for individuals with T2D, as reported by the Centers for Disease Control and Prevention (CDC) [16]. From 2000 to 2009, there was an age-adjusted decline in the incidence of DKA hospitalizations, with an average annual rate of change of -1.1%. However, the trend reversed during the period 2009-2014, with an increase in the rate from 19.5 to 30.2 per 1,000 persons, representing an average annual rate of 6.3% [21]. This reversal was observed across all age groups and both sexes, but the younger patients (<45 years old) had the highest rate of admissions. The explanation behind such higher DKA hospitalization rates is not univocal but rather depends on the combination of several reasons, such as changes in DKA definition as well as the widespread use of SGLT2 inhibitors and, ultimately, hospital admission of less severe cases. The incidence of DKA further increased during the Coronavirus disease (COVID-19) pandemic in individuals presenting with newly diagnosed T1D and in those with preexisting T2D [22, 23]. The exact mechanisms linking the onset of diabetes in those infected with the SARS-CoV-2 virus are yet to be fully elucidated. However, it is supposed that a number of complex, interrelated processes may be involved, including the detection of previously undiagnosed diabetes, stress hyperglycemia, steroid-induced hyperglycemia, and direct or indirect effects of severe acute respiratory syndrome coronavirus 2 on the beta cell [22-24]. A significant number of individuals hospitalized for DKA experience recurrent episodes, underscoring the importance of identifying potential triggers and preventing future occurrences [25]. A study conducted in Chicago between 2006 and 2012 found that 21.6% of individuals hospitalized for DKA had multiple episodes over six years [25]. Consistent with this observation, a 2014 analysis of inpatient data from the UK showed that 33.7% of individuals admitted for DKA had a history of at least one prior DKA episode within the previous year [26]. Generally, between 10% and 20% of patients are readmitted after an episode of DKA or hyperglycemic crisis. Of these readmissions, 40-65% are due to recurrent hyperglycemic crises, while the remaining cases may be attributed to other causes, occasionally including severe hypoglycemia. Most readmissions occur within two weeks following the patient's discharge from the previous DKA episode [27-29]. Hyperglycemic crises are linked to considerable morbidity, mortality, and costs [17, 30-32]. Conversely, DKA mortality showed a declining trend, with in-hospital case-fatality rates reduced from 1.1% to 0.4%, at an annual average rate of 6.8%, between the years 2000 to 2014, and the highest case-fatality rates were observed among patients older than 75 years. In summary, younger patients have a higher incidence of hospitalization and lower mortality rates. In contrast, older patients with multiple conditions have lower rates of hospitalizations but an elevated mortality risk associated with DKA [21]. After discharge from a DKA episode, individuals face a 13-fold increase in age-adjusted mortality within the following year [33]. Moreover, this discrepancy is more evident in younger subjects aged 15-39 years, with an increase in mortality rates up to 49 times as compared to the general population. In addition, when comparing patients with a single DKA admission to those with 2-5 admissions, the former group exhibits a threefold increased mortality risk, whereas the latter group has a sixfold increased risk [34]. In the USA, the estimated mean number of days spent in hospital by patients admitted with DKA is 3.0 days for individuals with T1D and 3.7 days for those with T2D [35]. In the UK, the mean hospitalization length is, on average, longer at 5.6 days [30]. Research in the USA estimates hospitalization costs for DKA patients to range from $21,215 to $36,600 per admission. These expenses are generally higher for individuals with T2D than for those with T1D and have been steadily rising over time [17, 27, 32]. In the UK, the estimated cost associated with a DKA admission was £ 2,064 per hospitalization [30]. These numbers highlight the significant economic burden DKA imposes on individuals and healthcare systems worldwide [36].

## 4 PATHOPHYSIOLOGY

**Fig. (1):**
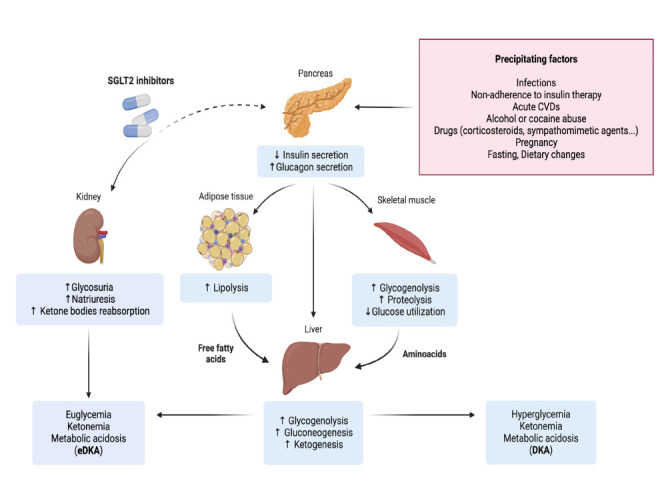
Pathophysiological mechanisms of DKA.

The primary pathophysiologic mechanisms underlying the development of DKA are complete or relative insulin deficiency and marked activation of counter-regulatory mechanisms resulting in increased serum glucagon, catecholamines, cortisol, and growth hormone [2]. In DKA, insulin deficiency may either be complete in subjects with T1D or partial, as observed in patients with T2D in the context of coexisting illnesses [16]. Higher catecholamines and cortisol levels promote protein catabolism and the release of amino acid precursors of gluconeogenesis. An imbalance in the glucagon/insulin ratio leads to gluconeogenesis and glycogenolysis through enzymatic stimulation in the liver while activating hormone-sensitive lipase in the adipose tissue. This enzymatic activation results in the breakdown of triglycerides into free fatty acids (FFA). Once in the hepatic mitochondria, FFA undergoes beta-oxidation, generating acetyl-coenzyme A, which is subsequently converted into ketone bodies due to citric acid cycle failure. The main ketone bodies that accumulate are acetoacetic acid and β-hydroxybutyrate, both strong acids. The dissociation of these acids produces a large number of hydrogen ions (H^+^) that are initially buffered by bicarbonate. However, the excessive production of H^+^ rapidly overwhelms the buffering capacity, leading to anion gap metabolic acidosis [37]. Because of insulinopenia, glucose internalization and utilization by peripheral tissues, particularly skeletal muscle, are impaired, thus enhancing hyperglycemia. When blood glucose levels exceed the renal threshold for glucose reabsorption (> 180 - 200 mg/dL), glycosuria appears. In turn, glycosuria raises intratubular osmolarity, triggering osmotic diuresis, which causes the loss of free water and electrolytes (including sodium and potassium), resulting in dehydration and hypovolemia. If this pathophysiological process persists, hypovolemia impairs glomerular filtration, reducing glycosuria and urinary excretion of ketoacids, thereby worsening metabolic acidosis and hyperglycemia [37]. Moreover, hypovolemia reduces peripheral tissue perfusion, causing lactic acidosis, with a detrimental effect on the existing acidosis. The coexistence of hyperglycemia, ketonemia, and acidosis represents the hallmark of DKA (Fig. **1**). However, exceptions exist: eDKA is a clinical triad comprising increased anion gap metabolic acidosis, ketonemia or ketonuria, and normal or minimally elevated blood glucose levels (<250 mg/dl) [38]. Several conditions are associated with eDKA, such as fasting, surgery, pregnancy, heavy alcohol consumption, illicit drug abuse, pancreatitis, insulin pump use, chronic liver disorders, and the use of SGLT2 inhibitors [39]. SGLT2 are sodium-dependent glucose transporters expressed in the proximal tubule that reabsorb up to 90% of the filtered glucose load. SGLT2 inhibitors are a new class of anti-hyperglycemic medications that are being used with increasing frequency in the treatment of DM [38, 40, 41]. SGLT2 inhibitors reduce glycemia primarily by enhancing glycosuria and decreasing the threshold for filtered glucose reabsorption. The reduction in serum glucose levels decreases insulin release and increases glucagon release from the pancreas. Furthermore, SGLT2 inhibitors appear to directly stimulate glucagon secretion from the pancreas. Finally, the high glucagon/insulin ratio promotes hepatic ketogenesis, as mentioned above. Hence, SGLT2 inhibitors are associated with a small but significantly increased risk of eDKA [38, 40, 42, 43]. In addition, certain medications can impact carbohydrate metabolism and also trigger DKA [44]. Glucocorticoids can cause both acute and sustained hyperglycemia by counteracting insulin action [45, 46]. Antipsychotic medications may also increase the risk of DKA, although the exact mechanism remains unclear [47].

In addition, recent evidence describes another DKA mechanism in the context of the so-called “Check-point Inhibitors Associated Diabetes Mellitus” or CIADM, a rare complication of immune check-point inhibitors (ICIs) therapy [48, 49]. Immune checkpoint inhibitors (ICIs) inhibit specific regulatory immune pathways such as programmed cell death-1 (PD1) and cytotoxic T-lymphocyte activating factor 4 (CTLA4) to enhance antitumor immune activity and are commonly used to treat several types of cancers. A consequence of ICIs use is the risk of triggering immune-related adverse events [48, 50, 51]. CIADM occurs secondary to autoimmune damage of pancreatic beta-cells and the incidence of DKA at presentation is 69.7%, usually occurring around 12 weeks after ICI commencement [48]. The traditional T1D autoantibodies are less prevalent in CIADM (40.4%) than in T1D (90%), with anti-GAD being the most frequently detected [48]. According to a recent meta-analysis, over 60% of life-threatening DKA occurring in CIADM were due to PD1 targeting agents, like pembrolizumab and nivolumab [52]. Nevertheless, understanding the complex immunology of CIADM remains challenging and necessitates the development of distinct diagnostic criteria and further research.

## 5 PRECIPITATING FACTORS

A wide range of factors can contribute to the start or precipitate the onset of DKA, among which infections are the most commonly observed (Fig. **1**). [26]. Other precipitating factors include discontinuation or inadequate insulin therapy, myocardial infarction, cerebrovascular accident, fasting, alcohol or cocaine abuse, and drugs that affect carbohydrate metabolism (corticosteroids, thiazides, sympathomimetic agents, pentamidine, *etc*.), as shown in Table **1** [53-55]. Notably, factors linked to an increased risk of hyperglycemic crises in individuals with T1D include younger age, a history of hyperglycemic or hypoglycemic events, kidney disease, neuropathy, depression, smoking, alcohol and substance abuse, elevated HbA1c levels, and social determinants of health (SDOH) [56-59]. Furthermore, individuals with diabetes and a history of DKA have a significantly higher prevalence of mental health disorders, including depression, diabetes distress, substance abuse, psychosis, and bipolar disorder, compared to those without a history of DKA [60, 61]. Psychological comorbidities, such as eating disorders, have been observed in recurrent DKA episodes, especially among young women with T1D. These psychological issues can lead to a fear of weight gain (*e.g.*, diabulimia), fear of hypoglycemia, rebellion against authority, stress of chronic disease, and, consequently insulin omission [61-63]. In individuals with T2D, risk factors encompass younger age, a history of hyperglycemic or hypoglycemic crises, comorbidities (both related and unrelated to diabetes), elevated HbA1c levels, and social determinants of health (SDOH) [34, 59, 62].

## 6 DIAGNOSIS

### 6.1 Clinical Presentation

Patients with DKA often present to the emergency department complaining of fatigue, nausea, vomiting, abdominal pain, polyuria, polydipsia, polyphagia, and weight loss [64]. On physical examination, signs of dehydration with dry mucous membranes, tachycardia, and low blood pressure are commonly observed and correlate with severe hypovolemia. In case of marked metabolic acidosis, Kussmaul breathing may develop, characterized by a classic fruity (acetone) breath odor. Altered mental status may also occur [16].

### 6.2 Laboratory Findings

The laboratory investigations are aimed primarily at diagnosis and to determine the severity of DKA. The biochemical profile should include a complete blood count, comprehensive metabolic panel, arterial or venous blood gas, and serum/urinary ketones. If clinically indicated, additional tests may be useful to rule out sepsis, ischemia, or other causes of acidosis, such as blood cultures, urinalysis, urine culture, electrocardiogram (ECG), cardiac biomarkers, chest x-ray, serum lactate, creatinine phosphokinase [4]. According to the recently published consensus report, the diagnosis of DKA can only be made when all three criteria (the “D,” the “K,” and the “A”) are present: blood glucose concentration of ≥ 11.0 mmol/L (200 mg/dL) or a prior history of DM, venous or capillary β-hydroxybutyrate concentration of ≥ 3.0 mmol/L or significant ketonuria (2+ or more on standard urine sticks), pH <7.3 and/or bicarbonate concentration of < 18.0 mmol/L [4]. The JBDS Guidelines propose a slightly different threshold, suggesting bicarbonate concentration <15 mmol/L instead of 18 mmol/L as part of the diagnostic criteria of DKA [3]. The nitroprusside reaction in urine or serum can be employed to perform a semiquantitative assessment of ketonemia. However, this method cannot quantify β-hydroxybutyrate, the main ketoacid produced in DKA. Alternatively, a quantitative assessment can be performed by directly measuring β-hydroxybutyrate in the blood, either through capillary point-of-care testing (POCT) or in a hospital laboratory setting [2]. Both ketones types demonstrate comparable diagnostic sensitivity; however, the measurement of serum β-hydroxybutyrate is more specific for the detection of DKA than the measurement of urinary acetoacetate [65]. If one relies only on urine, ketone testing may lead to an underestimation of ketonemia severity in the early stages of DKA, as acetoacetate forms later. On the other hand, it can cause an overestimation of severity in the later stages, when β-hydroxybutyrate is being cleared and converted into acetoacetate [2]. Moreover, a number of sulfhydryl drugs and other medications, including valproate, can result in a false-positive nitroprusside urine test result [4, 66]. Most people with DKA present with a high anion gap metabolic acidosis (>12 mmol/l). The anion gap is calculated by subtracting the major measured anions from the major measured cation: Na^+^ − [(Cl^−^ + HCO_3_^−^ (mEq/l). However, mixed acid-base disorders occur in about one-third of DKA cases due to hyperglycemia-induced osmotic diuresis and natriuresis, along with nausea and vomiting, which lead to volume contraction and the development of metabolic alkalosis. Additionally, a compensatory respiratory alkalosis may arise from hyperventilation, often caused by rapid or deep breathing patterns like Kussmaul's breathing [67, 68]. Furthermore, hyperchloremic normal anion gap acidosis is frequently observed following the successful treatment of DKA. It may result in a delay in the restart of subcutaneous insulin if it is misidentified as persistent DKA [69]. Depending on the extent of acidosis (blood pH, serum HCO3, and ketone levels) and the severity of mental status, DKA severity is classified as mild, moderate, or severe, as shown in Table **2** [4]. T1D can become clinically manifest through DKA in the pediatric population. The pathophysiology of DKA is similar in children and adults. Still, several pathophysiological and clinical features make it challenging to diagnose and manage in children promptly. Indeed, in the childhood and adolescent periods, the production of organic acids is significantly elevated in proportion to body weight, as are the basal metabolic rate and body surface area. Thus, it is more challenging to ascertain the grade of dehydration and to determine the correct fluid therapy. Moreover, clinical symptoms in children may be more ambiguous, requiring careful evaluation [53].

### 6.3 eDKA

In the absence of hyperglycemia, the diagnosis of eDKA only relies on pH level, serum bicarbonate, and ketonemia [42, 70, 71]. It is reported in young individuals with T1D, in pregnant women with pregestational T1D or T2D or gestational DM, in patients taking SGLT2 inhibitor therapy [72, 73]. The precipitating factors could be insulin omission or dose reduction, severe acute illness, cerebrovascular accidents, dehydration, low carbohydrate diets, excessive alcohol intake, cocaine abuse, and liver failure [74-77]. Moreover, a recent meta-analysis found that the presence of DM (either T1D or T2D) and the duration of disease >10 years are key determinants of an increased eDKA risk [78].

### 6.4 KPD

KPD patients are frequently middle-aged men (male: female ratio of approximately 3:1) of African American ethnicity with a positive family history and display a combination of overweight and dysmetabolic traits which closely resembles the clinical picture of classic T2D [79]. The disease typically manifests with severe hyperglycemia, severe ketosis, and, at times, with DKA, a presentation that immediately suggests T1D [79, 80]. Nevertheless, in contrast to other cases of DKA observed in individuals with T1D, in a considerable proportion of patients, autoantibodies to β-cells cannot be found. At presentation, both insulin production and action are severely impaired. However, aggressive insulin treatment can restore insulin production and action to levels comparable to T2D patients without DKA [81]. According to the AB classification scheme, the individuals with new-onset DM exhibiting DKA in the absence of IA2 and GAD65 autoantibodies (A^-^) and complete functional recovery of beta-cells (β^+^) were defined to suffer from A-β^+^ KPD. In contrast, those with DKA in the presence of IA2 and GAD65 autoantibodies (A^+^) but failure in functional recovery of beta-cells (β^-^) were defined as A^+^β^-^ KPD [82]. It has recently been reported that a state of near-normoglycemic remission correlates with enhanced recovery of both basal and stimulated insulin secretion. Furthermore, even 10 years after diabetes onset, approximately 40% of patients maintain partially preserved function of beta cells. Indeed, in this setting, fasting C-peptide levels > 1.0 ng/dl (0.33 nmol/l) and stimulated C-peptide levels > 1.5 ng/dl (0.5 nmol/l) have been identified as predictive of long-term normoglycemic remission in patients with a history of DKA [81].

## 7 MANAGEMENT

DKA is a medical emergency demanding timely diagnosis and intensive management. Mild to moderate DKA patients may be effectively treated in an emergency setting or step-down units [83-85]. Conversely, those presenting with severe DKA or a serious underlying illness as the precipitating cause require intensive care unit (ICU) admission [1, 59, 86]. The primary treatment objectives for DKA encompass resolving dehydration, correcting acidosis, ketosis, hyperglycemia, and electrolyte imbalances, and identifying/treating any precipitating factors.

### 7.1 Fluid Therapy

The primary and most crucial initial treatment for DKA is optimal fluid replacement, which aims to restore blood volume, improve renal perfusion, increase the urinary excretion of glucose and ketone bodies, and reduce insulin resistance by lowering circulating counter-regulatory hormone levels. In adults with DKA, the typical water deficit is around 100 ml/kg and should be corrected within 24-48 hours of diagnosis [87]. Isotonic saline (0.9% NaCl) is typically considered the optimal initial fluid replacement option due to its extensive availability, cost-effectiveness, and proven efficacy to re-establish circulating volume, as demonstrated in numerous clinical studies [59]. In the absence of cardiac or renal compromise, 0.9% NaCl should be administered at a rate of 500-1000 ml/h during the first 2-4 h [4]. It has been demonstrated that the administration of intravenous fluids in the absence of insulin results in a reduction in mean plasma glucose concentrations by approximately 2.8-3.9 mmol/l/h (50-70 mg/dl/h) [3]. Subsequent fluid replacement selection depends on various factors, including hemodynamics, hydration status, serum electrolyte levels, and urinary output. For patients with renal or cardiac compromise, careful monitoring of serum osmolality and regular assessment of cardiac, renal, and mental status are essential during fluid resuscitation to prevent iatrogenic fluid overload. Usually, within an interval of 4 to 8 hours, plasma glucose levels decline to <13.9 mmol/l (250 mg/dl) within a period of 4-8 hours, which precedes the resolution of DKA [88]. Therefore, upon reaching a plasma glucose concentration of <13.9 mmol/l (250 mg/dl). Therefore, in order to reduce the risk of hypoglycemia and guarantee insulin administration until the resolution of ketonemia, intravenous fluids administration should be adjusted to contain 5-10% dextrose in addition to 0.9% sodium chloride [59]. A cautious attitude is imperative for older adults with DKA, as alongside those with heart failure or end-stage kidney disease undergoing dialysis, proceeding with smaller boluses of isotonic or crystalloid solutions (*e.g.*, 250 ml boluses). It is crucial to assess their hemodynamic status to avoid complications frequently. Using standard fluid replacement protocols in these patients can lead to treatment-related issues such as volume overload, the necessity for mechanical ventilation, and extended hospital stays. Therefore, a tailored approach is essential to manage fluid therapy in these vulnerable populations [89].

### 7.2 Fluid Resuscitation: Balanced Electrolyte Solutions *versus* Isotonic Saline

There are two types of IV fluids commonly used for general fluid replacement in hospitalized patients:

Balanced Electrolyte Solutions (BES) are isotonic crystalloids that have a more similar composition to convalescent plasma in comparison to saline. It is typically used to replace fluid in dehydrated or volume-depleted patients. Examples of BES fluids commonly utilized in medical practice are Lactated Ringers (LR) solution and Plasma-Lyte 148 (PL).

Isotonic 0.9% NaCl is another isotonic crystalloid often used for fluid volume replacement.

Although isotonic 0.9% NaCl and BES are both isotonic, there are several differences between the two. For instance, 0.9% NaCl has a slightly higher osmolality (308 mOsm/L *vs*. LR 273 mOsm/L), lower pH (5.5 *vs* LR 6.6), and a higher chloride content (154 *vs* LR 109), which often correlates with the occurrence of hyperchloremic metabolic acidosis with 0.9% NaCl compared to BES [90, 91].

Current guidelines still suggest the use of 0.9% NaCl or isotonic saline infusion with pre-mixed potassium chloride as the preferred maintenance fluid in the management of DKA because it is compliant with the National Patient Safety Agency (NPSA) recommendations. Recently published metanalysis have shown that BES reverses DKA faster (in hours) than 0.9% NaCl [90, 92] and lowers the risk of hyperchloremia, in turn lowering acute kidney injury rates and metabolic acidosis [93]. Thus, BES is proposed as a superior alternative and has gained recent interest in the management of DKA [90].

### 7.3 Insulin Therapy

The pillar of DKA therapy is insulin administration, as it lowers blood glucose levels by reducing liver synthesis and enhancing its peripheral utilization. Additionally, insulin inhibits glucagon secretion, ketogenesis, and lipolysis, effectively reducing the synthesis of ketone bodies. Various healthcare institutions utilize different DKA treatment algorithms, employing varying units/kg/hour for insulin infusion. Continuous intravenous (IV) infusion of short-acting insulin is the preferred treatment. The available resources, as well as the severity of the patient clinical conditions, will drive the decision on whether to begin treatment through a fixed-rate intravenous insulin infusion, with a starting dose of 0.1 U/kg/h, or a nurse-driven protocol with a variable insulin infusion rate for DKA [1-3, 94]. Once blood glucose drops to 250 mg/dl (14 mmol/L), a 5-10% dextrose infusion should be added to 0.9% saline infusion and the IV insulin infusion rate could be lowered to 0.05 units/kg/h to mitigate the risk of hypoglycemia and hypokalemia [4]. Insulin and fluid adjustments should be made to maintain blood glucose levels at approximately 11.1 mmol/l (200 mg/dl) until resolution of DKA [2, 4, 87]. Although insulin is typically administered intravenously, multiple studies have shown that subcutaneous administration of rapid-acting insulin analogs every 1-2 hours can be a viable alternative to IV infusion for people with mild or moderate DKA [88, 95, 96]. A suitable regimen for subcutaneous insulin administration would be an initial bolus of rapid-acting insulin analog 0.1 U/kg given hourly until blood glucose drops below 250 mg/dl. After reaching this threshold, the dose is reduced to 0.1 U/kg every 2 hours or 0.05 U/kg given hourly until resolution of DKA [4, 53]. Despite similar clinical effectiveness, intramuscular injections of rapid-acting insulin this administration route is more painful than subcutaneous injections and may favour bleeding in patients receiving anticoagulation therapy [1, 97]. To note, for patients with arterial hypotension or those experiencing severe and complicated DKA, the utilization of rapid-acting subcutaneous insulin analogs is not recommended [53]. Maintenance of IV insulin administration for 1 to 2 h after the initiation of subcutaneous insulin is instrumental in preventing the recurrence of hyperglycemia or ketosis during the transition to subcutaneous treatment. Interestingly in this concern, implementing practice-based advisory systems has shown efficiency to reduce the risk rebound DKA [98]. Individuals who were previously on insulin should resume their usual insulin dosage, with adjustments as necessary. If there are concerns about insufficient baseline insulin therapy or any drug that may have contributed to the DKA event, the treatment plan should be revised at discharge rather than postponed to outpatient follow-up [1, 59]. Insulin-naive patients should start with long-acting analog subcutaneously at 0.15-0.3 U/kg. This medication can be administered either once daily or divided into two equal doses, given twice daily. Rapid-acting insulin is introduced as needed based on nutritional intake and glucose levels [4]. The concurrent administration of basal insulin and a fixed-rate intravenous insulin infusion is a practice that is endorsed by numerous clinicians despite the fact that some avoid it due to concerns about the potential for hypoglycemia [99] and hypokalemia [100]. Research indicates that adding a low dose (0.15-0.3 U/kg) of basal insulin during the infusion can reduce the time needed to resolve DKA, shorten the duration of insulin infusion, and decrease the length of hospital stay [101, 102]. It also helps prevent rebound hyperglycemia, all without increasing the risk of hypoglycemia [101, 103]. The first necessary step in order to transition from IV to subcutaneous insulin is the calculation of the total daily insulin requirement. The estimated total daily dose (TDD) of insulin can be estimated using a number of different methods: first, a weight-based formula using 0.5-0.6 U/kg/day in insulin-naïve patients. However, it is important to consider that the aforementioned estimate may be influenced by factors such as body composition and insulin resistance. Likewise, for individuals who are at risk for hypoglycemia, such as those with frailty or reduced kidney function, a calculation using around 0.3 U/kg/day may be more suitable [4]. Secondly, evaluating the insulin regimen and disease control before hospital admission can assist in determining the appropriate transition dosing requirements. Nevertheless, it is imperative to evaluate the potential impact of patient adherence to medication regimens and dietary habits on the recommended outpatient insulin dosing regimens. Lastly, the TDD of insulin may be evaluated by taking into account the hourly intravenous insulin infusion requirements. However, this should be approached cautiously due to potential variations in insulin needs influenced by factors such as glucotoxicity, duration of intravenous insulin treatment, concurrent dextrose infusion, medications that affect blood sugar levels, and nutritional intake [4]. A basal-bolus insulin regimen, using basal and rapid-acting insulin analogs, is considered a more physiological approach. This regimen has been shown to reduce the incidence of hypoglycemia after transitioning from intravenous to subcutaneous insulin following DKA resolution, compared to using human insulins (such as short-acting and NPH insulins) [88]. Currently, there are no studies available on transitioning to ultra-long-acting insulins (*e.g.*, degludec, glargine U300).

### 7.4 Electrolyte Imbalance Correction

DKA manifests various electrolyte abnormalities due to the elevated extracellular osmolarity resulting from high blood glucose levels, causing an intracellular water shift to the extracellular space and diluting extracellular sodium. This effect is balanced by osmotic diuresis, where water loss exceeds sodium loss. In adults with DKA, the total body sodium deficit typically ranges from 7-10 mmol/kg [87]. Due to excess extracellular water, pseudo-hyponatremia is a common encounter at admission.. Corrected serum sodium is usually calculated by accounting for hyperglycemia levels to evaluate the severity of sodium and fluid deficits [26]. Hypokalemia and hyperkalemia are both critical and commonly observed in DKA, as metabolic acidosis and insulin deficiency promote the movement of potassium into the extracellular space [104]. Consequently, despite an initial appearance of normal potassium concentration, total body potassium is actually low.

The estimated potassium deficit in DKA adults is around 3-5 mmol/kg [2]. Insulin therapy lowers serum potassium levels by promoting its shift back into the intracellular compartment. Besides insulin therapy, factors such as acidosis correction, volume repletion, and kaliuresis also contribute to the reduction in serum potassium. Notably, within the first 48 hours of treatment, potassium levels typically drop by 1-2 mmol/L [26]. Potassium replacement should be promptly started after serum potassium levels fall below 5.0 mmol/L, to prevent hypokalemia and maintain a steady potassium level of 4-5 mmol/L [3, 59]. Consensus reports recommend adding 20-30 mmol of potassium per liter of intravenous fluid to preserve target serum potassium concentrations [4]. With serum potassium levels below 3.5 mmol/L, it is crucial to replenish potassium at a rate of 10 mmol/h before starting insulin infusion to prevent insulin-induced hypokalemia caused by intracellular potassium shifting. In this case, insulin administration should be postponed until potassium levels reach >3.5 mmol/L to minimize the risk of arrhythmias and respiratory failure [105]. The consensus report recommends assessment of serum potassium from 2 h after starting insulin therapy and every 4 h thereafter until the resolution of DKA to avoid hypokalaemia [4].

### 7.5 Bicarbonate Administration

Routinary use of bicarbonate is not advised due to the likelihood that appropriate fluid and insulin therapy will effectively solve the acidosis in DKA, particularly when arterial pH is above 7.0 [104, 106]. Additionally, excessive bicarbonate can elevate the CO_2_ partial pressure in cerebrospinal fluid and potentially trigger paradoxical acidosis in the central nervous system (CNS), cerebral oedema, and hypokalaemia. Consequently, bicarbonate therapy should be considered for patients with a pH below 7 and those with heart failure. Guideline recommendations suggest administering a sodium bicarbonate infusion of 100 mmol (mEq) in 400 ml of sterile water volume over 2 hours, continuing until the pH > 7 [59, 106].

### 7.6 Phosphate Deficiency

Upon admission, serum phosphate concentration is frequently within normal or elevated levels. However, in adults with DKA, the body often experiences a phosphate deficit estimated to be approximately 1 mmol/kg due to intracellular phosphate shifting into the extracellular space with a loss of phosphate in the urine, resulting in hypophosphatemia [107]. Severe phosphate deficiency can lead to rhabdomyolysis, arrhythmias, and respiratory failure. Nevertheless, in the absence of evidence of physical symptoms related to hypophosphatemia (namely muscular weakness, respiratory fatigue, or cardiac impairment) and phosphate levels below 1.0 mmol/L, the routine replacement is unwarranted [4]. Whenever clinically appropriated, 20-30 mEq of phosphate can be supplemented per liter of infused solution, all while carefully monitoring calcium levels to prevent tetany [4, 53]. A number of prospective randomized studies have yielded inconclusive results with regard to the impact of phosphate replacement on the clinical outcome of DKA [2, 108]. Furthermore, there is evidence to suggest that excessively rapid phosphate supplementation may induce hypocalcemia [108].

### 7.7 eDKA

eDKA is defined as DKA with a blood glucose level below 200 mg/dL (11.1 mmol/L) in the presence of ketosis and metabolic acidosis criteria of DKA [42, 70, 71]. eDKA is found among patients with DKA in up to 10% of subjects. It is most frequently encountered in those experiencing low glucose conditions, such as infections, pregnancy, starvation, alcohol use disorder and chronic liver disease [42]. Additionally, eDKA has been linked to the use of SGLT2 inhibitors, which enhance urinary glucose excretion [38, 42, 71]. As a result, insulin resistance, decreased insulin secretion, and limited glucose availability predispose patients to ketogenesis, potentially leading to eDKA. Management of this condition is similar to other forms of DKA but must account for the absence of hyperglycemia. Treatment includes resuscitation with intravenous fluids, insulin, glucose, and addressing the underlying precipitating. In contrast to DKA management, where blood glucose levels are typically higher, eDKA presents with glucose levels less than 200 mg/dL (11.1 mmol/L). Therefore, it is crucial to initiate dextrose 5-10% immediately at a rate of 125 ml/hr. Following initial fluid replacement, continuous intravenous insulin infusion should commence at a rate of 0.1 units/kg/hr. If glucose levels continue to fall despite the 10% glucose infusion, reduce the insulin rate to 0.05 units/kg/hr to prevent hypoglycemia, maintaining a target serum glucose level of 150-200 mg/dL [87]. Close monitoring of serum electrolytes and glucose levels is essential throughout the treatment. If DKA occurs with SGLT2 inhibitors use, they should be stopped on admission. Whenever an alternative aetiology of eDKA is identified and/or solved, the resumption of SGLT2 inhibitor is eventually recommended. SGLT2 inhibitor therapy is not recommended for patients with T1D. In patients with T2D, initiation or continuation of SGLT2 inhibitor therapy after DKA resolution is not routinely recommended [4, 109]. The criteria for resolution of DKA are purely biochemical and include: a) plasma ketone < 0.6 mmol/l; b) serum bicarbonate level ≥ 18 mEq/l; c) venous pH ≥ 7.3 d) ideally, blood glucose < 200 mg/dL (11.2 mmol/L) [4]. The anionic gap should not serve as a criterion as this can lead to misinterpretation due to hyperchloremic acidosis resulting from a high volume of 0.9% sodium chloride. Also, as β-hydroxybutyrate is converted to acetoacetate as the acidosis improves, the measurement of ketones in the urine should be avoided as a criterion for the resolution of DKA [4].

## 8 COMPLICATIONS

DKA triggers a proinflammatory response and promotes blood coagulation, increasing the risk of thrombosis, especially when central venous catheters are employed [53]. In the absence of thrombosis, a prophylactic dose of low-molecular-weight heparin is recommended as a means of mitigating the risk of thrombosis [4]. Transient acute kidney injury may occur in up to 50% of adults [87, 110]. This condition is more commonly observed in older adults, individuals with elevated osmolarity, and those presenting with high glucose levels at admission. Acute kidney injury typically resolves with adequate rehydration. Daily monitoring of renal function is advised [4].

1. Pulmonary oedema is an uncommon complication of DKA, typically seen in patients with pre-existing heart disease or following an overly rapid infusion of crystalloids [87].
2. Excessive correction of high blood glucose levels with insulin, leading to hypoglycemia (<2.2 mmol/l or < 40 mg/dl), may trigger the secretion of counter-regulatory hormones, resulting in acidosis, cardiac arrhythmias, acute brain damage, and potentially, death. This can be prevented by monitoring blood glucose every 1-2 h, reducing the insulin infusion rate to 0.05 U/kg/h when glucose levels are reduced to < 13.9 mmol/l (250 mg/dl) and initiating a 5 - 10% dextrose infusion alongside a 0.9% NaCl solution [4, 87].
3. Hypokalemia is a common complication resulting from intracellular potassium shifts after insulin treatment. Hypokalaemia (<3.5 mmol/l) occurred in ~55% of DKA. Severe hypokalaemia <2.5 mmol/l occurs in 16% of people with DKA and it is associated with increased inpatient mortality. This can be prevented by monitoring kalaemia every 4 h during treatment and adding potassium to fluid resuscitation [4, 26].
4. Cerebral oedema is rare in adults but can be a complication of DKA in children. It represents a significant complication with a documented mortality rate of approximately 30% in comparison to those without oedema [111]. The risk of cerebral oedema seems to be higher in younger individuals and those recently diagnosed with DM [112]. The exact cause of this phenomenon is unknown. The risk factors that predispose individuals to cerebral oedema should lead to an expansion of either the intracellular or extracellular fluid compartments of the brain. It is evident that if one of these intracranial compartments were to expand without a corresponding decrease in the volume of the other, it would elevate intracranial pressure due to the brain's confinement within the skull. Two significant factors have the potential to increase the intracerebral intracellular fluid (ICF) volume: an increase in the number of solute molecules confined within cells (effective osmoles) and a reduction in the effective P_osm_, due to the rapid movement of water across cell membranes to achieve effective osmotic equilibrium. Some of these risk factors exert an early influence, whereas others exert their principal effects at a later stage of therapy for DKA. The presence of cerebral oedema in nearly 5% of patients before DKA therapy strongly indicates the existence of pre-existing risk factors. It has been suggested that one such factor could be a less restrictive blood-brain barrier (BBB) upon admission in DKA patients. Therefore, a bolus of saline solution could increase the volume of the intracranial interstitial space. This could be attributed to two primary factors. Firstly, capillary hydrostatic pressure may elevate due to expanded plasma volume. Secondly, a decrease in plasma albumin concentration due to dilution might result in a drop in colloid osmotic pressure. Additionally, the net valence on plasma proteins directly influences colloid osmotic pressure and it has been demonstrated a significant decrease in the net anionic charge on plasma proteins following a rapid saline infusion. A bolus of insulin could expand the intracerebral intracellular fluid volume by converting the inactive form of the Na+/H+ exchanger (NHE) to its active form and this causes Na+ to enter and H+ to exit from cells [112]. Increased H+ concentration in the ICF compartment is another activating factor for NHE. This leads to an increase in the number of solute molecules in the ICF, as most of the exported H+ were primarily bound to ICF proteins or entered cells alongside β-hydroxybutyrate through the monocarboxylic acid transporter. Given the usual presence of intracellular acidosis in DKA patients and the existence of insulin receptors in the brain an early intravenous insulin bolus could potentially have a more significant intracerebral effect, especially when the BBB might be less restrictive to insulin passage. One of the risk factors for cerebral oedema later in DKA therapy is a swift decrease in blood glucose levels (P_Glu_), which could subsequently lower the effective plasma osmolality (P_osm_) unless there is a corresponding appropriate increase in plasma sodium levels (P_Na_). The P_Glu_ usually falls by 5 mmol/l/h over the initial six hours of therapy. It is necessary to increase the P_Na_ in order to prevent a decline in the effective P_osm_ when there is a reduction in P_Glu_. The P_Na _must be >140 mmol/l if the P_Na_ on admission is close to 140 mmol/l [31]. In conclusion, a constant infusion rather than a bolus of insulin should be given as the only seconds to minutes emergency that is reversed by insulin is life-threatening hyperkalemia. Only in cases of severe hypotension should 0.9% NaCl solution be rapidly infused. 0.9% NaCl is the preferred intravenous solution for the first day because the aim is to prevent effective P_osm_ falling. While asymptomatic cerebral oedema may frequently occur, vigilance is crucial, particularly when unexpected neurological deterioration or prolonged coma without an apparent cause is observed. Consequently, any decline in the Glasgow Coma Scale score should trigger urgent medical intervention and prompt imaging. In this concern, promising horizons are revealed through advanced imaging techniques, that offer the chance to characterize residual brain function better and guide neurological recovery care [113]. If cerebral oedema is suspected and fulfills clinical diagnostic criteria, immediate treatment with mannitol or hypertonic saline should be initiated without delay due to neuroradiological confirmation [31]. Mannitol dose is 0.5 - 1 g/kg in 10 - 15 min; instead, hypertonic saline (3%) dosages are 2.5 - 5 ml/kg in 10- 15 min, in alternative or in addition to mannitol or if it isn't effective in 15 - 30 min [53].

## 9 PREVENTION

Although they focus on the vast majority of aspects related to DKA identification and management, the existing guidelines still lack comprehensive measures for the prevention of DKA, particularly for the management of ketosis without acidosis, with or without severe hyperglycemia. This review underscores the need for common strategies to manage such conditions both at home and in hospital settings to prevent the development of DKA. Research indicates that children diagnosed with T1D often have multiple physician visits in the days or weeks leading up to their diagnosis, especially among very young children [114].

Regular awareness campaigns for physicians about diabetes early symptoms and the need for urgent treatment, especially in very young patients, should be implemented periodically [115, 116].

In this concern, a recent systematic review found that awareness campaigns reduced DKA rates by 1% to 65.5%, with the variation largely influenced by the unique features of each campaign. These efforts were linked to fewer acute complications, lower HbA1c at diagnosis, higher C-peptide levels, and shorter hospital stays. Notably, the costs of these campaigns were significantly lower than the expenses associated with treating DKA cases [117].

As noted earlier, a significant number of individuals hospitalized for DKA may experience recurrent episodes. Approximately 10% to 20% of patients are readmitted to the hospital following an episode of acute diabetes complication. The occurrence of recurrent episodes of DKA is associated with an elevated risk of long-term cognitive decline and premature mortality [25]. Contributing factors include insulin omission, socioeconomic disadvantage, and mental illness [53, 118]. It would be advisable to implement a series of strategies with the aim of preventing episodes of DKA. The most crucial of the strategies that can be employed is the education of patients on the significance of glucose monitoring, including the recognition of individual glycemic targets and the identification of factors that may precipitate significant glycemic variations outside of the normal range. The educational programme should include a review of injection techniques and urine or blood ketone testing, as well as the administration of appropriate adjustments to insulin therapy. To prevent episodes of DKA and ensure the achievement of recommended glycemic targets, it is imperative to implement comprehensive glucose monitoring. It has been demonstrated that continuous glucose monitoring (CGM) is more effective than self-monitoring of blood glucose (SMBG) in achieving optimal glycemic control and reducing hospitalizations due to acute diabetes complications [119, 120]. The results of the RELIEF study demonstrated that two years after the introduction of CGM, the rate of hospitalizations for patients with T1D and T2D declined by 49% and 48%, respectively [120]. This improvement is largely due to a significant decrease in DKA episodes.

Current methods for ketone testing, such as capillary blood and urine testing, have limitations. Emerging technologies like continuous ketone monitoring (CKM) use subcutaneous sensors to update ketone levels every few minutes. These devices evaluate interstitial fluid by means of a subcutaneously inserted sensor employing enzymatic electrochemical reactions (in a way like CGM). In the very next future, it is supposed the development of devices with alarms linked to standardized thresholds and trend indicators, aiming to reduce user burden. Ideally, CKM functionality should be integrated with other devices used for CGM and insulin pumps. Recording ketone ranges, CKM may indicate conditions of urgency, suggesting practical responses to the proposed display of ketone data, both in real-time and as a retrospective review. Potential beneficiaries include those with T1D, pregnant individuals, users of medications like SGLT2-inhibitors that increase DKA risk, individuals prone to recurrent DKA, socially or geographically isolated individuals, or those following low carbohydrate diets during hospitalization or during “sick days.” Providing ketone profiles will offer critical clinical insights previously unavailable to people with diabetes and their healthcare providers [121].

In recent decades, innovative technologies such as CGM, insulin pumps, and automated insulin delivery (AID) systems have been introduced to enhance T1D management. These technological advancements aim to achieve better glycemic control and reduce the occurrence of acute complications, including severe secondary DKA and severe hypoglycemia. For example, the Control-IQ Observational (CLIO) Prospective Study evaluated 3,157 participants in the United States, reporting reduced adverse event rates, including DKA, among users of the T:slim X2™ insulin pump equipped with Control-IQ technology [122].

Further preventive strategies may include access to telemedicine and digital communication tools, the availability of a 24-hour emergency response service, and timely follow-up within two to four weeks after discharge. Additional risk factors for recurrent episodes include adolescence and psychiatric disorders such as depression, diabulimia, and schizophrenia [118]. Therefore, it would be beneficial to employ an integrated approach in clinical practice, which would also encompass psychological and social support.

## CONCLUSION

Despite significant advances in diagnostic and therapeutic approaches, diabetic ketoacidosis (DKA) remains a serious complication of diabetes mellitus (DM). It poses a threat both at the onset of type 1 diabetes (T1D) and during periods of acute metabolic disturbance in both T1D and type 2 diabetes (T2D).

Currently, clinical guidelines for DKA management are primarily based on expert opinions and consensus rather than comprehensive outcome-driven research. There is a pressing need for large-scale randomized controlled trials and well-designed observational studies across diverse populations. These studies should focus on: 1) optimizing the electrolyte composition of intravenous fluids, 2) identifying the best insulin administration rates and methods; and 3) establishing an optimal potassium replacement regimen.

Furthermore, more research is necessary to develop strategies that prevent recurrent DKA episodes, particularly in high-risk individuals. A holistic approach involving psychological support, telemedicine advancements, and the education of family members and caregivers has proven effective in reducing DKA occurrences. Prospective studies should target individuals facing mental health challenges, diabetes-related distress, and depression [114]. Continuous Glucose Monitoring (CGM) systems have demonstrated superiority over capillary glucose monitoring in achieving optimal glycemic control in both T1D and T2D patients. A nationwide study conducted in France found that access to CGM was associated with a 53% reduction in DKA hospitalizations for T1D and a 47% reduction for T2D [117]. Although CGM has not yet been approved for hospital use in DKA management, it is recommended for patients post-discharge [118]. Emerging Continuous Ketone Monitoring (CKM) technologies hold promise for further improving metabolic control and reducing user burden, providing real-time updates on ketone levels. A novel approach for youth with poorly controlled T1D involves using a pump to deliver rapid-acting insulin for boluses alongside a once-daily subcutaneous injection of long-acting insulin. This hybrid strategy can offer the full benefits of pump therapy while reducing DKA risk without compromising glycemic control. This method is particularly beneficial for those hesitant to use a pump continuously due to body image concerns or frequent physical activities [123].

Lastly, public awareness campaigns, alongside physician education and patient screening efforts, are essential for equipping families and healthcare professionals with the tools to recognize early warning signs, seek timely medical care, and adopt effective diabetes management practices [124].

In summary, preventing DKA requires coordinated educational campaigns, technological innovations, and targeted research efforts to advance diagnostic methods and management strategies. International collaborations and studies are crucial to mitigate this life-threatening complication and improve the lives of individuals living with diabetes.

## Figures and Tables

**Table 1:** DKA precipitating factors in adult and in children.

Precipitating factors in adult DKA	Precipitating factors in children DKA
Poor compliance with insulin therapy or anti-diabetic treatment	New onset of T1D
Acute cardiovascular events (myocardial infarction, stroke, pulmonary embolism)	Poor compliance with insulin therapy
Infection	Insulin error
Alcohol or cocaine abuse	Insufficient education and inadequate technical handling of the pump
Underlying illness	Infection
Pregnancy	-
Dietary changes (low carbohydrate diet, pre/post operative fasting)	-

**Note:** Adapted from Lapolla *et al.* [54].

**Table 2:** Diagnostic criteria for DKA.

** Diagnostic criteria for DKA **	-	-	-	-
-	-	**Mild**	**Moderate**	**Severe**
**D:** blood glucose concentration	Glucose ≥ 11.0 mmol/L (200 mg/dL) or known to have DM	≥ 11.1 mmol/l (200 mg/dL)	≥ 11.1 mmol/l (200 mg/dL)	≥ 11.1 mmol/l (200 mg/dL)
**K:** ketones	β-Hydroxybutyrate blood concentration ≥ 3.0 mmol/L or ketonuria (urine ketone strip 2+ or more)	β-Hydroxybutyrate 3.0-6.0 mmol/l	β-Hydroxybutyrate 3.0-6.0 mmol/l	β-Hydroxybutyrate >6.0 mmol/l
**A:** acidosis	pH <7.3 and/or serum bicarbonate concentration <18 mmol/L	pH >7.25 to <7.30 or bicarbonate 15-18 mmol/l	pH 7.0-7.25 or bicarbonate 10 to <15 mmol/l	pH <7.0 or bicarbonate <10 mmol/l
**Mental status**	-	Alert	Alert/drowsy	Stupor/coma

**Note:** Adapted from Umpierrez *et al.* [4].

## LIST OF ABBREVIATIONS

DKA: Diabetic Ketoacidosis
DM: Diabets Mellitus
eDKA: Euglycemic DKA
T1D: Type 1 Diabetes
T2D: Type 2 Diabetes
ADA: American Diabetes Association
EASD: European Association for the Study of Diabetes
JBDS: Joint British Diabetes Societies for Inpatient Care
AACE: American Association of Clinical Endocrinology
DTS: Diabetes Technology Society
FDA: Food and Drug Amministration
EMA: European Medicines Agency
KPD: ketosis-prone Diabetes
FFA: Free Fatty Acids
SDOH: Social Determinants Health
CIADM: Check-point Inhibitors Associated Diabetes Mellitus
ICIs: Immune Checkpoint Inhibitors
PD1: programmed Cell Death-1
CTLA4: Cytotoxic T-lymphocyte Activating Factor 4
POCT: Cpillary Point-of-care Testing
ICU: Intensive Care Unit
BES: Balanced Electrolyte Solutions
LR: Lactate Ringer
IV: Intravenous
TDD: Total Daily Dose
CNS: Central Nervous System
ICF: Intracerebral Intracellular Fluid
BBB: Blood-Brain Barrier
NHE: Na+/H+ Exchanger
CGM: Continuous Glucose Monitoring
AID: automated insulin delivery
CLIO: Control-IQ Observationa Prospective Study
